# Relationship between immune‐related adverse events and treatment effectiveness in extensive disease small cell lung cancer

**DOI:** 10.1111/1759-7714.15010

**Published:** 2023-06-26

**Authors:** Keiki Yokoo, Yauo Kitamura, Keito Suzuki, Kohei Morikawa, Takeo Sawai, Hiroyuki Honda, Sayaka Kudo, Gen Yamada

**Affiliations:** ^1^ Department of Respiratory Medicine Teine Keijinkai Hospital Sapporo Japan; ^2^ Department of Respiratory Medicine Kushiro City General Hospital Kushiro Japan; ^3^ Department of Respiratory Medicine Hakodate Goryoukaku Hospital Hakodate Japan; ^4^ Department of Pulmonary Medicine Steel Memorial Muroran Hospital Muroran Japan; ^5^ Nagasaki University Hospital Infectious Diseases Experts Training Center Nagasaki Japan

**Keywords:** extensive disease small cell lung cancer, immune checkpoint inhibitors, immune‐related adverse events, irAEs

## Abstract

**Background:**

This study aimed to assess the relationship between immune response adverse events (irAEs) and treatment efficacy in patients with extensive disease small cell lung cancer (ED‐SCLC).

**Methods:**

We retrospectively evaluated the clinical effects in 40 ED‐SCLC patients who had received immune‐checkpoint inhibitors (ICIs), platinum agents, and etoposide between September 2019 and September 2021. We identified and compared patients belonging to two groups: irAE and non‐irAE.

**Results:**

Fifteen patients experienced irAEs, and 25 did not. The median progression‐free survival in patients with irAE was longer than that in patients without irAE (12.6 months [95% CI: 6.3–19.3 months] vs. 7.2 months [95% CI: 5.8–7.9 months], *p* = 0.0108). However, the median overall survival (OS) was similar between irAE and non‐irAE groups (27.6 months [95% CI: 15.4–NA] vs. 24.9 months [95% CI: 13.7–NA], *p* = 0.268). Seven (46.7%) in the irAE group and 20 (80%) in the non‐irAE group received sequential therapy. The median OS was prolonged in patients who received first‐ and second‐line therapy than in those who received first‐line therapy alone (27.6 months [95% CI: 19.2–NA] vs. 6.6 months [95% CI: 0.3–NA], *p* = 0.053). Grade ≧ 3 irAEs occurred in five (12.5%) patients. Among them, grade 5 irAEs were observed in two patients, including exacerbation of polymyositis and pulmonary arterial embolism.

**Conclusion:**

In this study, the development of irAEs did not affect OS in patients with ED‐SCLC who received platinum‐based agents, etoposide, or ICI therapy. We determined that managing irAEs and administering first‐ and second‐line therapies could contribute to prolonged OS.

## Graphical abstract

In extensive disease small cell lung cancer, immune‐related adverse events were less associated with prolonged overall survival.

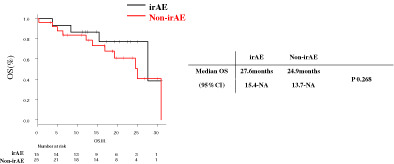

## INTRODUCTION

Small cell lung cancer (SCLC) accounts for approximately 15% of all lung cancers. About two‐thirds of SCLC patients have extensive disease (ED) at diagnosis. Two clinical trials, the IMpower133 and CASPIAN studies, have demonstrated survival benefits in patients with ED‐SCLC.^1^, ^2^

In the IMpower133 trial, atezolizumab alongside carboplatin and etoposide therapy demonstrated significantly extended survival (median overall survival [OS] 12.3 months; 95% confidence interval [CI]: 10.8–15.8) than carboplatin and etoposide therapy (median OS 10.3 months; 95% CI: 9.3–11.3).^2^ In the CASPIAN trial, durvalumab alongside a platinum agent and etoposide therapy demonstrated significantly enhanced survival (median OS 12.9 months; 95% CI: 11.3–14.7) than a platinum agent and etoposide therapy (median OS 10.5 months; 95% CI: 9.3–11.2) and 3‐year survival rate (17.6% vs. 5.8%).^3^

Based on these findings, immune checkpoint inhibitors (ICIs), platinum agents, and etoposide are considered mainstream therapies. However, the survival benefits of adding ICIs to cytotoxic agents in ED‐SCLC are numerically lower than those in non‐small cell lung cancer (NSCLC).^4^, ^5^ Moreover, it is unclear whether the occurrence of immune‐related adverse events (irAEs) is related to the response, progression‐free survival (PFS), and OS in SCLC. Although some reports suggest a strong correlation between the occurrence of irAEs and survival in NSCLC,^6^ there are no reports on the relationship between irAEs and the combination of chemotherapy and ICI therapy in ED‐SCLC, except for ICI monotherapy and combination therapy.^7^ Interestingly, in patients treated with nivolumab, memory T cell was detected to bind nivolumab in the blood more than 20 weeks after the final dose. Thus, even more than six months after the final administration, caring for irAEs is essential.^8^

This study aimed to retrospectively elucidate the relationship between irAEs, clinical efficacy, and survival benefits.

## METHODS

### Study design and patients

This retrospective study included ED‐SCLC patients who received first‐line therapy between September 2019 and September 2021 at two institutions in Hokkaido, Japan (Teine Keijinkai Hospital and Kushiro City General Hospital).

The eligibility criteria were as follows: pathologically diagnosed ED‐SCLC or small recurrence after chemoradiation therapy. All patients underwent systematic evaluation using computed tomography (CT), brain magnetic resonance imaging (MRI), and bone scintigraphy or 18F‐fluorodeoxyglucose positron emission tomography before first‐line therapy. Patients who relapsed more than 6 months after the last cytotoxic agent administration were enrolled. IrAEs were assessed between the day of the initial ICI administration and six months after the final ICI administration.

This study was approved by the Institutional Review Board of Teine Keijinkai Hospital (no. 2‐022484‐00). All procedures complied with the ethical standards of the institutional and/or national research committee and the 1964 Declaration of Helsinki and its subsequent amendments or comparable ethical standards. As this was a retrospective study, the requirement for informed consent was waived.

### Statistical analysis

Statistical analyses were performed using Fisher's exact test and Welch's *t*‐test for categorical and continuous variables, respectively. The chi‐square test was used to compare the proportions of patients based on their characteristics (sex, performance status [PS], and smoking). PFS was defined as the time interval between initial treatment administration and disease progression or death from any cause. OS was defined as the time interval between the initial treatment administration and any cause of death or censored on the date of the last follow‐up. Patients without documented clinical or radiographic disease progression or those still alive were censored on the date of the last follow‐up.

PFS and OS were evaluated using the Kaplan–Meier method and compared using a two‐sided log‐rank test. Univariate analyses using Cox proportional hazards modeling were performed to determine the correlation between OS and various factors. Hazard ratio (HR) and 95% CI were estimated using the Cox proportional hazards regression model. All *p*‐values were two‐tailed, and the threshold for statistical significance was set at *p* < 0.05.

All statistical analyses were performed using EZR version 1.40 (Saitama Medical Center, Jichi Medical University, Saitama, Japan).

## RESULTS

### Patient characteristics

**FIGURE 1:**
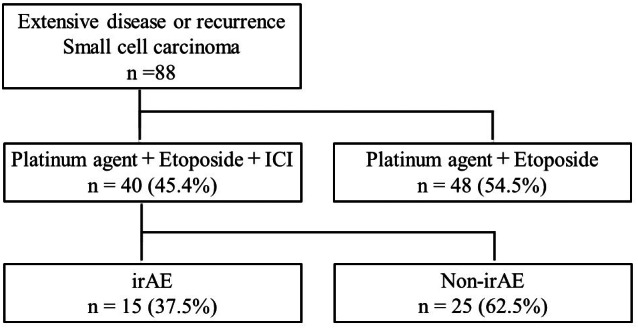
Consort diagram of this study.

A total of 88 patients were enrolled in this study between September 2019 and September 2022 (Figure 1). Of these patients, 40 received platinum agents, carboplatin or cisplatin, etoposide, ICI therapy, atezolizumab, or durvalumab. In contrast, 48 patients received a platinum‐based agent and etoposide, not ICI therapy, due to interstitial pneumonia, poor PS, and advanced age. IrAE occurred in only 15 patients (37.5%; “irAE” group) but not in 25 (62.5%) (“non‐irAE” group).

The baseline demographics and disease characteristics of the patients are summarized in Table 1. Thirty of the ICI‐receiving patients (75%) were male, all of whom smoked, and 37 (92.5%) had extensive stage disease. The median age at the initiation of first‐line treatment was 69 years (range, 53–77 years) in the irAE group and 70 years (range, 49–78 years) in the non‐irAE group. PS was well‐balanced between the two groups. There were seven (46.7%) patients with metastatic lesions at more than two sites in the irAE group and 12 (48%) in the non‐irAE group. The neutrophil‐lymphocyte ratio (NLR) was 4.2 (range 1.7–8.7) in irAE and 3.3 (range 1.2–14.3) in the non‐irAE group. Furthermore, five patients did not experience progressive disease (PD) events.

**TABLE 1:** Baseline and treatment characteristics.

Characteristics	No (%)			
	All (*n* = 40)	With irAE (*n* = 15)	Without irAE (*n* = 25)	*p*‐value
Age (years)				
Median (range)	69.5 (49−78)	69 (53−77)	70 (49−78)	0.84
Sex				0.45
Male	30 (75)	10 (66.6)	20 (80)	
Female	10 (25)	5 (33.3)	5 (20)	
Smoking status				1.0
Current or former/never	40 (0)	15 (0)	25 (0)	
Stage				1.0
Extensive stage	37 (92.5)	14 (93.3)	23 (92)	
Recurrence	3 (7.5)	1 (6.7)	2 (8)	
Performance status (ECOG)				1.0
0	5 (12.5)	2 (13.3)	3 (12)	
1	34 (85)	13 (86.7)	21 (84)	
2	1 (2.5)	0 (0)	1 (4)	
Number of metastatic organs				0.94
<2	21 (52.5)	8 (53.3)	13 (52)	
≥2	19 (47.5)	7 (46.7)	12 (48)	
Intracranial metastases at entry				1.0
Yes	11 (27.5)	4 (26.7)	7 (28)	
No	29 (72.5)	11 (73.3)	18 (72)	
Liver metastases at entry				0.69
Yes	8 (20)	2 (13.3)	6 (24)	
No	32 (80)	13 (86.7)	19 (76)	
Pleural effusion or pleural metastases at entry				0.715
Yes	13 (32.5)	6 (40)	7 (28)	
No	27 (67.5)	9 (60)	18 (72)	
Neutrophil count (/μL), median (range)	4915 (2450−14 370)	5500 (2580−13 490)	4890 (2450−14 370)	0.98
Lymphocyte count (%), median (range)	19.2 (6.0−39.5)	17 (10.1−32.3)	19.5 (6−39.5)	0.62
NLR, median (range)	3.65 (1.2−14.3)	4.2 (1.7−8.7)	3.3 (1.2−14.3)	0.89
Alb (g/dL), median (range)	3.7 (1.8−4.5)	3.7 (2.9−4.4)	3.5 (1.8−4.5)	0.46
Type of ICI				
Atezolizumab	28	10	18	0.74
Durvalumab	12	5	7	
ICI maintenance therapy				
Yes/No	33/7	12/3	21/4	1.0
Number of cycles of ICI maintenance therapy, median	4	4	4	
Reason for discontinuation of ICI				
Progression disease	26 (78.8)	5 (50)	21 (91.3)	<0.01
Adverse events	4 (12.1)	4 (40)	0 (0)	
Worsened performance status	2 (6.1)	1 (10)	1 (4.3)	
Others	1 (3.0)	0 (0)	1 (4.3)	
Sequential therapy (Yes/No)	27/13	7/8	20/5	<0.05
Amrubicin	14 (51.9)	3 (42.9)	11 (55)	
Topotecan	7 (25.9)	1 (14.3)	6 (30)	
CBDCA + ETP	2 (7.4)	0 (0)	2 (10)	
Irinotecan	1 (3.7)	0 (0)	1 (5)	
Beyond atezolizumab	2 (7.4)	2 (28.6)	0 (0)	
Others	1 (3.7)	1 (14.3)	0 (0)	

Abbreviations: Alb, albumin; CBDCA, carboplatin; ETP, etoposide; ICI, immune‐checkpoint inhibitor; irAE, immune‐related adverse event; NLR, neutrophil‐to‐lymphocyte ratio.

A total of 27 (67.5%) patients received sequential therapy. Amrubicin was the most commonly administered drug (51.9%). Two patients received ICIs beyond PD after local therapy because the progressive lesion was in a single organ.

### Efficacy

**FIGURE 2:**
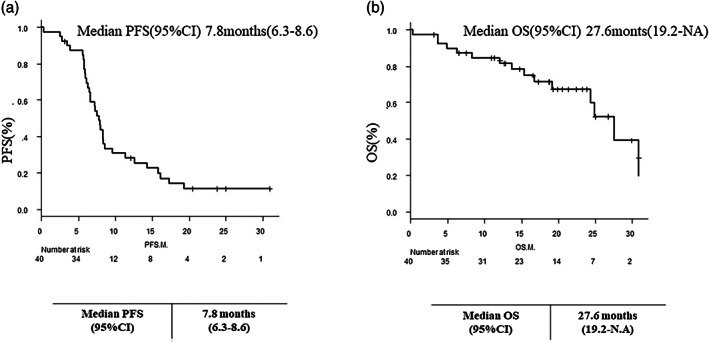
Kaplan–Meier curves of (a) progression‐free survival and (b) overall survival of all patients receiving a platinum agent and etoposide and ICI therapy. ICI, immune checkpoint inhibitor; M, month; OS, overall survival; PFS, progression‐free survival.

The response and disease control rates in the overall population were 87.5 and 97.5%, respectively. The response rates for irAEs and non‐irAEs were 93.3 and 84%, respectively (Table 2). The median follow‐up period in the overall population was 12.1 months (range, 0.3–30.9), the median progression‐free survival was 7.8 months (range, 6.3–8.6), and the OS was 27.6 months (range, 19.2–NA) (Figure 2a,b).

**TABLE 2:** Best tumor response to first‐line treatment.

Tumor response	No. (%)		
	ALL	With irAE	Without irAE
	(*n* = 40)	(*n* = 15)	(*n* = 25)
ORR	35 (87.5)	14 (93.3)	21 (84)
DCR	39 (97.5)	15 (100)	24 (96)
CR	4 (10)	4 (26.7)	0 (0)
PR	31 (77.5)	10 (66.7)	21 (84)
SD	4 (10)	1 (6.7)	3 (12)
PD	1 (2.5)	0 (0)	1 (4)

Abbreviations: CR, complete response; DCR, disease control rate; irAE, immune‐related adverse events; ORR, objective response rate; PD, progressive disease; PR, partial response; SD, stable disease.

**FIGURE 3:**
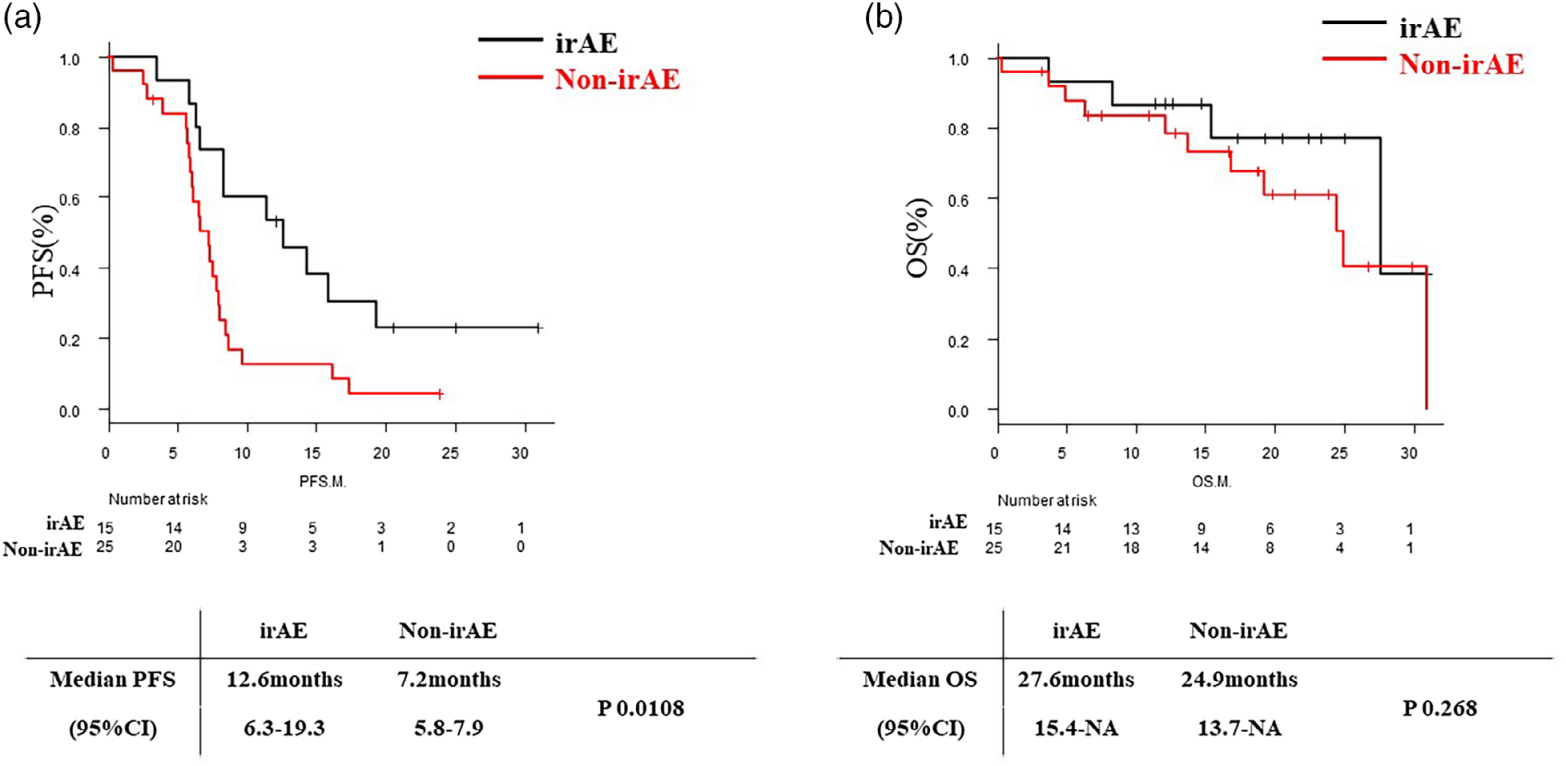
Kaplan–Meier curves of (a) progression‐free survival and (b) overall survival of patients with irAE and non‐irAE. ICI, immune checkpoint inhibitor; M, month; OS, overall survival; PFS, progression‐free survival.

The irAE group had a longer PFS (12.6 months; 95% CI: 6.3–19.3 months) than the non‐irAE group (7.2 months; 95% CI: 5.8–7.9 months) (*p* = 0.0108). The median overall survival was 27.6 months (95% CI: 15.4–NA) in irAE and 24.9 months (95% CI: 13.7–NA) in non‐irAE (*p* = 0.268) (Figure 3a,b). Regarding NLR, a threshold of 5 did not affect PFS or OS (Figure S1).

**FIGURE 4:**
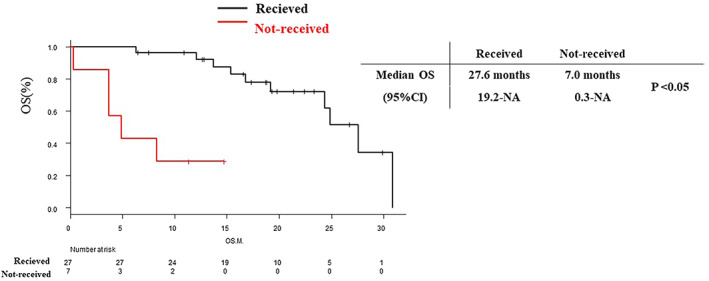
Kaplan–Meier curves of overall survival of patients who received or did not receive (excluded non‐PD patients) subsequential therapy. ICI, immune checkpoint inhibitor; M, month; OS, overall survival; PFS, progression‐free survival.

In the population that received subsequent therapy, excluding non‐PD patients, the median OS was 27.6 months (95% CI: 19.2–NA). Conversely, the median OS was 6.6 months (95% CI: 0.3–NA) in patients who did not receive subsequent therapy (Figure 4).

We performed univariate analysis using Cox proportional hazards modeling for OS (Table S1). Due to the number of OS events being 15, we did not attempt a multivariate analysis. The factors evaluated were intracranial metastasis, liver metastasis, PS, irAEs (with or without) and irAEs (≥grade 3). With regard to PS, analysis was performed for patients with PS 0 and 1, and not for patients with PS 2 as there was only one patient. No independent factors were identified for OS.

### Toxicity

Treatment‐related AEs are shown in Table 3, and irAEs in Table 4. The most common treatment‐related AEs were anemia (80%), decreased white blood cell (77.5%) and neutrophil (72.5%) counts. Grade 3–4 AE of decreased neutrophil count and febrile neutropenia occurred in 28 (70%) and two (5%) patients, respectively. Grade 5 AE of pulmonary arterial emboli occurred in one patient (2.5%).

**TABLE 3:** Treatment‐related adverse events (AEs).

Adverse events	No. (%)					
	Any grade	Grade 1	Grade 2	Grade 3	Grade 4	Grade 5
White blood cell count decreased	31 (77.5)	4 (10)	6 (15)	14 (35)	7 (17.5)	0 (0)
Neutrophil count decreased	29 (72.5)	0 (0)	1 (2.5)	3 (7.5)	25 (62.5)	0 (0)
Febrile neutropenia	2 (5)	0 (0)	0 (0)	2 (5)	0 (0)	0 (0)
Anemia	32 (80)	11 (27.5)	15 (37.5)	6 (15)	0 (0)	0 (0)
Platelet count decreased	19 (47.5)	5 (12.5)	4 (10)	8 (20)	2 (5)	0 (0)
Aspartate aminotransferase increased	12 (30)	10 (25)	0 (0)	2 (5)	0 (0)	0 (0)
Alanine aminotransferase increased	11 (27.5)	9 (22.5)	0 (0)	2 (5)	0 (0)	0 (0)
Creatinine kinase increased	7 (17.5)	4 (10)	1 (2.5)	1 (2.5)	1 (2.5)	0 (0)
Lung infection	2 (5)	0 (0)	2 (5)	0 (0)	0 (0)	0 (0)
Herpes zoster	2 (5)	0 (0)	0 (0)	2 (5)	0 (0)	0 (0)
Thromboembolic event	1 (2.5)	0 (0)	0 (0)	0 (0)	0 (0)	1 (2.5)

**TABLE 4:** Immune‐related adverse events (irAEs).

Immune‐related adverse events	No. (%)					
	Any grade	Grade 1	Grade 2	Grade 3	Grade 4	Grade 5
Rash	8 (20)	3 (7.5)	5 (12.5)	0 (0)	0 (0)	0 (0)
Thyroid disorder	6 (15)	4 (10)	2 (5)	0 (0)	0 (0)	0 (0)
Adrenal disorder	3 (7.5)	3 (7.5)	0 (0)	0 (0)	0 (0)	0 (0)
Pituitary dysfunction	1 (2.5)	0 (0)	1 (2.5)	0 (0)	0 (0)	0 (0)
Pneumonitis	2 (5)	0 (0)	2 (5)	0 (0)	0 (0)	0 (0)
Colitis	2 (5)	0 (0)	0 (0)	2 (5)	0 (0)	0 (0)
Diabetic ketoacidosis	1 (2.5)	0 (0)	0 (0)	0 (0)	1 (2.5)	0 (0)
Sclerosing cholangitis	1 (2.5)	0 (0)	1 (2.5)	0 (0)	1 (2.5)	0 (0)
Exacerbation of connective tissue disease	2 (5)	0 (0)	1 (2.5)	0 (0)	0 (0)	1 (2.5)

The most common irAE was skin disorders (20%). Grade 4 irAEs of diabetic ketoacidosis and sclerosing cholangitis occurred in one patient (2.5%). Grade 5 irAEs of connective tissue disease exacerbation occurred in one patient (2.5%).

## DISCUSSION

To our knowledge, this is the first study to report that irAEs caused by durvalumab and atezolizumab affected the survival of patients with ED‐SCLC. In this retrospective observational study for ED‐SCLC, the median PFS was 12.6 months (95% CI: 6.3–19.3 months) in the irAE group, and 7.2 months (95% CI: 5.8–7.9 months) in the non‐irAE group, with there being a significant difference. In addition, the median OS was 27.6 months (95% CI: 15.4–NA) and 24.9 months (95% CI: 13.7–NA) in the irAE and non‐irAE groups, respectively. This study aimed to elucidate the relationship between irAEs and treatment efficacy. The reinvigorated immune cells due to ICI treatment attack not only tumor cells but also normal tissue, and it has been previously reported that the occurrence of irAEs could lead to a better response.^9^ However, our study showed that irAEs were less associated with prolonged OS and PFS than non‐irAEs. Although the characteristics and eligibility criteria differed between our study and the clinical trials, the longer OS in our study is notable.

In this study, the median OS of 27.6 months (range: 19.2–NA) in the population was better than clinical trials; 12.3 months in the IMpower133^2^ and 12.9 months in the CASPIAN studies.^3^ Similarly, a subgroup analysis of the Japanese population in the IMpower133 study showed a better trend than that of the global population.^10^

The longer OS observed in our study was thought to be due to the better management of irAEs and the ratio of subsequent therapies. In this study, 40 patients received a platinum‐based agent, etoposide, ICI, atezolizumab, or durvalumab, and IrAEs developed in 15 patients (37.5%). A total of 35 patients discontinued first‐line treatment because of PD and irAEs. Of these patients, 27 (77.1%) received second‐line therapy. A more significant proportion of patients received subsequent therapy in this study than in clinical trials: 50.2% in the IMpower 133 study^2^ and 46% in the CASPIAN study.^3^ Moreover, this was similar to the values of the Japanese subgroup analysis: 65% in the IMpower 133 study^10^ and 76% in the CASPIAN study.^11^ However, 20 patients (80%) in the non‐irAE group received subsequent therapy, compared to only seven (46.7%) in the irAE group.

These findings suggest that managing irAEs in ED‐SCLC is more difficult in practice than in previous reports.^10^, ^11^ In this study, the rate of grade 3 or higher irAEs was 12.5%, consistent with previous reports.^10^, ^11^ However, one patient developed a grade 5 AE, pulmonary arterial emboli, and another developed a grade 5 irAE and exacerbation of connective tissue disease. There are various types of irAEs ranging from easy to manage, difficult, and life‐threatening. Lethal irAEs such as myocarditis and neuromuscular disorders are rare. However, it is important to identify and manage irAEs immediately.

Although both irAE and non‐irAE groups underwent a median of four courses of maintenance therapy, the median PFS was longer in the irAE group than in the non‐irAE group. Regarding the onset of irAEs, the median time from the start of ICI therapy to the onset of irAEs was 4.9 months (range 0.4–20.1). It appears that in some cases the tumor continued to shrink despite discontinuation of ICI therapy for the successful management of irAEs. We believe those reasons contributed to prolonged PFS.

In this study, recurrent SCLC patients were defined as patients who had received prior chemoradiotherapy for limited‐stage SCLC and had a treatment‐free interval of at least 6 months since last chemoradiotherapy, according to the protocol of the IMpower 133 study. Three patients were enrolled and grade 2 irAEs that were manageable had developed in one patient. Median OS was 29.9 months which was longer than in all patients. Although the number of patients with recurrent SCLC was small, it appear that patients with recurrent SCLC were tolerant to chemotherapy along with ICI therapy.

Regarding NLR, due to lack of data about patients with ED small, we established a threshold value of 5 on the basis of previously published data.^12^ However, that threshold value did not affect PFS or OS. Moreover, we analyzed other threshold values of 2 and 3. Both values had no effect on OS (data not shown). Although there was a limited number of cases, our results demonstrated that NLR could not be a prognostic factor for OS in ED‐SCLC.

Although no phase 3 study previously evaluated the efficacy of amrubicin in combination with platinum doublet and ICI therapy in ED‐SCLC, a study reported a median OS of 11.7 months in sensitive relapse and 7.3 months in refractory relapse after treatment with atezolizumab plus carboplatin and etoposide.^13^ These data indicate that amrubicin is an effective and feasible second‐line treatment option. Furthermore, in our study, the median OS of patients receiving second‐line therapy was prolonged compared to those who did not (Figure 4). Based on these findings, administering second‐line therapy after controlling irAEs may contribute to prolonged OS in ED‐SCLC.

Our study had some limitations. First, it was a retrospective study with a small number of patients. Physicians at each institution selected eligible patients. For example, in one institution, patients who demonstrated positivity for antibodies, such as collagen vascular disease, without physical findings, such as seropositivity, did not receive ICI therapy plus chemotherapy as the first‐line treatment. In addition, due to the limited number of cases, we could not perform a multivariate analysis, and a larger cohort study is therefore needed.

Second, the skills required to identify irAEs depend on the physicians. The assessment and immediate action of irAEs are important for subsequent therapy. However, there is a possibility that physicians could not immediately identify irAEs, and the ratio of patients receiving therapy was low for irAEs. These biases and differences among physicians may have affected the results of our study; however, we believe that our results are valuable in practice.

In conclusion, the development of irAEs did not affect the OS in ED‐SCLC patients who received platinum agents, etoposide, or ICI therapy. Immediate management of irAEs could be attributed to the increased ratio of subsequent therapy and prolonged OS.

## AUTHOR CONTRIBUTIONS

Keiki Yokoo, Yauo Kitamura, Gen Yamada developed the concept, designed the study, interpreted the results, and drafted the manuscript. All authors collected the data and approved the results and conclusions.

## CONFLICT OF INTEREST STATEMENT

The authors have no conflicts of interest to declare.

## Supporting information


**Figure S1.** Kaplan–Meier curves of overall survival of patients with NLR <5 or NLR ≥5. ICI, immune checkpoint inhibitor; M, month; OS, overall survival; PFS, progression‐free survival.

**Table S1.** Univariate analyses of overall survival.
