# Supplementary figures and images for: Relationship between immune‐related adverse events and treatment effectiveness in extensive disease small cell lung cancer
Source: Thorac Cancer. 2023 Jun 26;14(23):2251–8. doi: 10.1111/1759-7714.15010 (PMC10423651; doi:10.1111/1759-7714.15010)

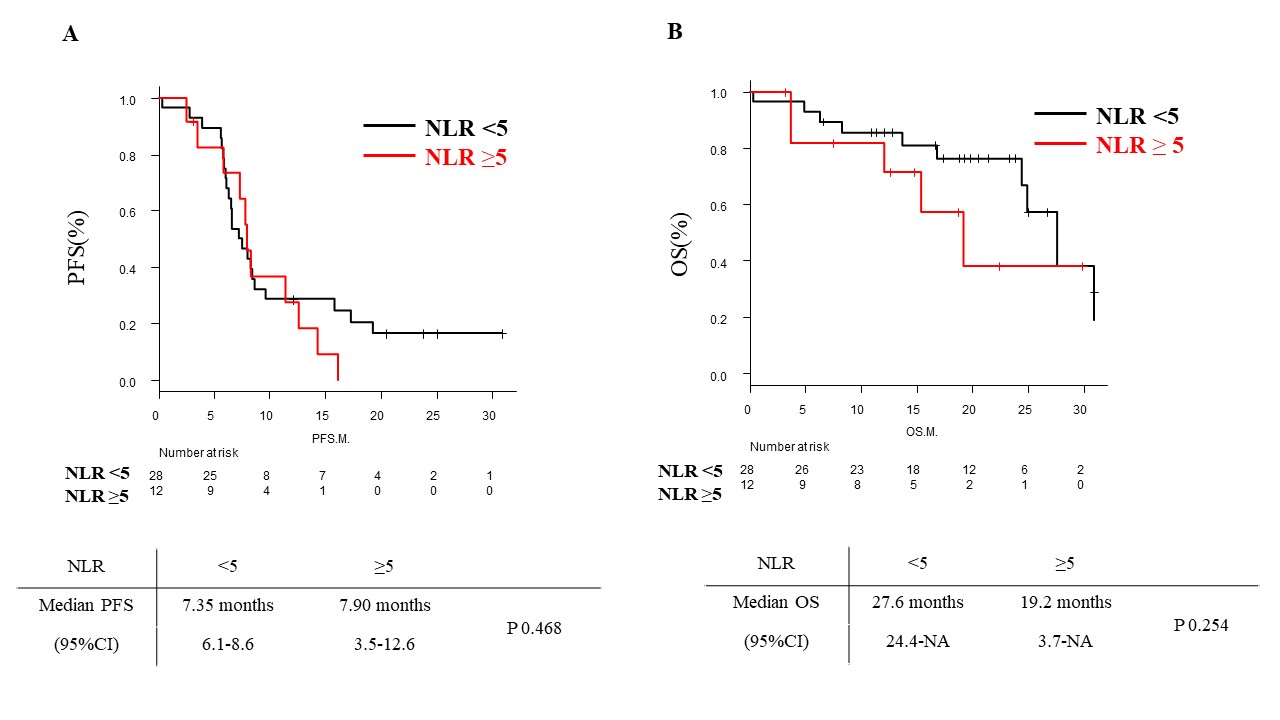

Supplement: Supplementary file 1 — Figure S1. Kaplan–Meier curves of overall survival of patients with NLR <5 or NLR ≥5. ICI, immune checkpoint inhibitor; M, month; OS, overall survival; PFS, progression‐free survival. [file TCA-14-2251-s001.jpg]
