# Supplementary material for: Relationship between immune‐related adverse events and treatment effectiveness in extensive disease small cell lung cancer
Source: Thorac Cancer. 2023 Jun 26;14(23):2251–8. doi: 10.1111/1759-7714.15010 (PMC10423651; doi:10.1111/1759-7714.15010)
Supplement: Supplementary file 2 — Table S1. Univariate analyses of overall survival. [file TCA-14-2251-s002.docx]

**TABLE S1. 　Univariate analyses of overall survival**

| **Variable** | | | | HR | 95% CI | p |
| --- | --- | --- | --- | --- | --- | --- |
| **Brain metastasis** | **yes** | ***v*** | **no** | 1.35 | (0.35-5.12) | 0.65 |
| **Liver metastasis** | **yes** | ***v*** | **no** | 1.48 | (0.41-5.37) | 0.54 |
| **irAEs** | **yes** | ***v*** | **no** | 0.53 | (0.17-1.67) | 0.28 |
| **irAEs (**≥**Grade3)** | **yes** | ***v*** | **no** | 0.69 | (0.09-5.33) | 0.72 |
| **Performance Status**† | **1** | ***v*** | **0** | 1.26 | (0.27-5.93) | 0..76 |

| Abbreviations: | CI, confidence interval; HR, hazard ratio; irAE, immune-related adverse event; PS, performance status |
| --- | --- |
| † Analyzed performed patients with PS 0 and 1 due to only one patient with PS2. | |
